# Postexercise Impact of Ice-Cold Water Bath on the Oxidant-Antioxidant Balance in Healthy Men

**DOI:** 10.1155/2015/706141

**Published:** 2015-03-19

**Authors:** Paweł Sutkowy, Alina Woźniak, Tomasz Boraczyński, Celestyna Mila-Kierzenkowska, Michał Boraczyński

**Affiliations:** ^1^The Chair of Medical Biology, Nicolaus Copernicus University, Ludwik Rydygier Collegium Medicum in Bydgoszcz, 24 Karlowicza Street, 85-092 Bydgoszcz, Poland; ^2^Central Research Laboratory, Józef Rusiecki Olsztyn University College, 33 Bydgoska Street, 10-243 Olsztyn, Poland

## Abstract

The aim of the study was to determine the effect of a 5 min head-out ice-cold water bath on the oxidant-antioxidant balance in response to exercise. The crossover study included the subjects (*n* = 24; aged 28.7 ± 7.3 years) who performed two identical stationary cycling bouts for 30 min and recovered for 10 min at room temperature (RT = 20°C; session 1) or in a pool with ice-cold water (ICW = 3°C, 5 min immersion; session 2). The concentration of thiobarbituric acid reactive substances (TBARS) in blood plasma (TBARSpl) and erythrocytes (TBARSer) and the erythrocytic activity of superoxide dismutase (SOD), catalase (CAT), and glutathione peroxidase (GPx) were measured three times during each of the two study sessions: before the exercise (baseline) and 20 and 40 min after the appropriate recovery session. Lower concentration of TBARSpl 40 min after postexercise recovery in ICW was revealed as compared with that after recovery at RT (*P* < 0.05). Moreover, a statistically significant postexercise increase in the TBARSpl and TBARSer concentrations was found (*P* < 0.01 and *P* < 0.05, resp.). A short-term ice-cold water bath decreases postexercise lipid peroxidation.

## 1. Introduction

Low temperatures can induce different effects in the human body depending on the value, as well as duration and type of exposure. The effects of moderate but long-term cold during winter season (meaning daily temperature below 0°C for several months), found in people living in high latitude environments (circumpolar residents), are definitely negative. Among the mildest effects are unpleasant sensations and thermal discomfort. The more serious effects are decreased physical and mental performance resulting in an increased risk of accidents and injuries, as well as an increase in morbidity or even mortality at very low temperatures [1]. However, exposure to cold is widely used in sports medicine for accelerating recovery and improving sports performance. This is due to the positive effects obtained by using extremely low or moderately low temperatures at an adequately short period [2]. The most popular methods of cold therapy in sports, due to their anti-inflammatory/antiedematous and analgesic properties, are ice compresses (crushed ice packs), cold-water immersion (CWI) baths (8–10°C for 4-5 min), and whole-body cryotherapy (WBC) sessions (the vapor of liquid synthetic air at a temperature between –100 and –160°C for maximum 3 min) [2]. Specifically, CWI and ice compresses have been effectively employed for this purpose because of their effectiveness and low expense [2–4]. Another type of cold stimulation, ice-cold water (ICW) bathing, also known as winter swimming in countries where waters freeze during wintertime, has similar properties as the aforementioned cold therapies [5]. Therefore, it is used as a treatment method in rheumatic diseases [5]. Usually, during ICW baths, water temperature is 0–4°C, air temperature is below or a few degrees above 0°C, and duration is between tens of seconds and a few minutes [5–7]. Although ICW baths affect the human organism similarly as the already mentioned cold therapies and are much cheaper than, for example, WBC sessions, no exact protocol has proven better than the other [8, 9]. Moreover, the literature indicates that ICW baths may not even offer any real benefit and, in fact, may increase postexercise muscle soreness the day after intense exercise [10]. Therefore, the rationale for using ice-cold water baths to assist the recovery of performance and the determination of their optimal protocols require further studies. Moreover, studies of the effects of low temperatures on the oxidant-antioxidant balance related to recovery/performance have been conducted only with the use of WBC [2, 11–13]. The authors emphasize that WBC sessions modulate that balance in a way that improves sports performance [11–13]. Therefore, in the studied subjects' blood, the concentration of thiobarbituric acid reactive substances (TBARS) and the activity of superoxide dismutase (SOD), catalase (CAT), and glutathione peroxidase (GPx) were measured. TBARS are markers of the lipid peroxidation process [11], while the three enzymes are the most important antioxidant agents and markers of an increased generation of reactive oxygen species (ROS) in the human organism [12]. TBARS and the mentioned enzymes have been accepted by many authors as markers of the disturbance in the oxidant-antioxidant balance induced by exercise and environmental extremes [12–15].

The aim of the study was to investigate the effect of a 5 min ice-cold water bath on the postexercise oxidant-antioxidant balance in healthy men.

## 2. Materials and Methods

### 2.1. Subjects

24 healthy men aged 28.7 ± 7.3 years volunteered for the study. The subjects had never performed winter swimming or bathing in ice-cold water before the study. The subjects did not change their eating habits or physical activity immediately before or during the study period. The subjects' characteristics are presented in Table 1. The levels of physical activity and aerobic capacity were similar for all subjects.

The study was approved by the Bioethics Committee at Nicolaus Copernicus University, Ludwik Rydygier Collegium Medicum in Bydgoszcz (Poland) (number KB 657/2012). The subjects were informed about the study aims and the potential risks associated with the study and signed informed consent forms.

### 2.2. Study Overview

The study was divided into 2 sessions with several subjects per week for 1.5 months (a total of 24 subjects). On Mondays (session 1), the subjects performed stationary cycling for 30 min (single submaximal physical exertion), whereupon they recovered in a gym in a sitting position for 10 min (room temperature, RT, 20°C). On Fridays (session 2), the subjects performed the same single 30 min exercise bout and then recovered in a small swimming pool with ice-cold water (ICW, a single 5 min immersion at 3°C; total time: 10 min including undressing). The study subjects were dressed only in swimming trunks and immersed their whole bodies except their heads.

The study was of a crossover design; that is, the subjects were randomly assigned either to RT or ICW conditions for session 1, while for session 2 the same subjects switched their previous recovery conditions. The temperatures were measured with an accuracy of 0.2°C. Moreover, the temperature of ice-cold water was achieved and remained consistent using crushed ice.

During both sessions, blood for laboratory assays was taken from the basilic vein to 9 mL “Vacuette EDTA” tubes 3 times: directly before the cycling bout (baseline), as well as 20 min and 40 min directly after the recovery at RT or in ICW. The laboratory analyses were conducted no later than a few hours after the samples had been taken. During that time, blood was transported to the laboratory in a refrigerator at 4°C and the subsequent centrifugation of the samples in the laboratory was performed at the same temperature.

### 2.3. Outcomes

An analysis of the body composition of the study subjects, using the Tanita body composition analyzer (Tanita BC 418 MA Corporation, Japan) and the Bioelectric Impedance Analysis (BIA) technique, was performed. Selected indexes are presented in the paper: body mass (BM, kg), the percentage of body fat (BF, %), body height (BH, cm), total body water (TBW, %), muscle mass (MM, kg), body mass index (BMI, kg/m^2^), and body surface (BS, m^2^) (Table 1).

For rating perceived exertion (RPE), the Borg Category-Ratio-10 (CR10) scale was used [16]. The first rate “0” means “no exertion at all.” In turn, the last rate on the scale, marked as “10,” means “extremely strong” effort. There is also an exertion rate over 10, marked as “∗.” This is an exertion that makes the subject “unable to continue” an exercise bout. The RPE scale was used in both study sessions after both 30 min exercise bouts (Table 1). The subjects assessed the exercise bouts as “moderate/somewhat hard” [16].

The International Physical Activity Questionnaire (IPAQ) was used to assess the level of physical activity of the subjects during the last 7 days before the study [17]. The IPAQ is expressed in MET·min/week. One MET = 3.5 mL O_2_/min/kg and represents the baseline oxygen consumption. The level of physical activity of the study subjects was moderate (2574.3 ± 1000.6; Table 1). This means that the subjects met at least one of the following 3 criteria: (i) 3 or more days of vigorous activity of at least 20 minutes per day, (ii) 5 or more days of moderate-intensity activity or walking of at least 30 minutes per day, and (iii) 5 or more days of any combination of walking, moderate-intensity, or high-intensity activities achieving a minimum of at least 600 MET·min per week [17].

In order to determine the aerobic fitness of the study subjects, the maximum oxygen consumption (VO_2max⁡_) was assessed directly before the 30 min exercise bouts using the physical working capacity-170 (PWC_170_) test as an indirect method [18, 19]. The PWC_170_ index means the load (watts, W) for which heart rate (HR) amounts to 170 beats per minute (bpm). The basis for determining the PWC_170_ index is the 10 min standard cycle ergometer test during which the load in the second half is increased in such a way not to exceed the HR of 170 bpm. The PWC_170_ index was determined using the following linear function: PWC_170_ =* P*_1_ + (*P*_2_  −* P*_1_)/(170 − HR_1_)(HR_2_  − HR_1_), where* P*_1,2_ = loads of the first and the second half of the test and HR_1,2_ = heart rates during the first and the second half of the test [18, 19]. The value of PWC_170_ index is significantly correlated with the value of VO_2max⁡_; therefore, the VO_2max⁡_ variables of all the subjects were calculated using the values of PWC_170_ test according to the Astrand-Ryhming nomogram [20]. The values of VO_2max⁡_ are presented in Table 1. They demonstrate that the aerobic fitness of the study subjects was average [20]. There is also a linear relationship between the power/load of exertion and HR between 120 bpm and 170 bpm for men aged 19 to 40; therefore, intensities and loads of the 30 min submaximal exercise bouts were determined individually for each subject using the PWC_170_ index via a recalculation to the PWC_140_ index [18, 19]. Analogously, the PWC_140_ index means a load generating the HR of 140 bpm. The mean power of the first exercise bout (session 1) was 153.3 ± 41.2 W to 78.8 ± 17.8% of maximum heart rate (HR_max⁡_) and the mean power of the second exercise bout (session 2) was 169.4 ± 49.5 W to 79.5 ± 7.5% of HR_max⁡_ (HR_max⁡_ = 205 − (age/2)). There were no statistically significant differences between the loads, HRs, VO_2max⁡_ values, and Borg CR10 variables; thus, the two exercise bouts were the same. However, all of these parameters were determined separately for each of the two exercise trials to ensure that they are the same, although the subjects were asked to maintain their eating habits, physical activity, and other lifestyle factors immediately before and during the study period. Earlier, the authors' own pilot studies (not published) revealed that some volunteers at an age similar to that of the subjects in this study were unable to finish the 30 min exercise bout defined by the PWC_170_ index; therefore, the PWC_140_ index was used.

Blood samples were centrifuged for 10 min at 6000 ×g at 4°C. After the centrifugation, the upper layer (plasma) was removed. Subsequently, the isolated cells were washed three times with a phosphate-buffered saline (PBS) solution at a ratio of 1 : 3 with a simultaneous centrifugation of the sample after each wash (6000 ×g/10 min). During the removal of the supernatant, the top layer of white blood cells and platelets was removed as well. Monitoring of the presence of protein in the supernatant was conducted with a 20% aqueous solution of sulfosalicylic acid, and when the reaction was negative, the washed red blood cells were mixed with a PBS solution in such way to obtain erythrocytic suspension with 50% of hematocrit index. A total of five tests were done per one blood sample: one per blood plasma (TBARSpl) and four per erythrocytic suspension (TBARSer, CAT, SOD, and GPx).

The TBARS concentration was determined in both the blood plasma (TBARSpl) and erythrocytes (TBARSer) using the spectrophotometric method by Buege and Aust [21], which was modified by Esterbauer and Cheeseman [22]. To 0.5 mL of erythrocytic suspension or plasma, 4.5 mL of reaction mixture containing 0.375% thiobarbituric acid (TBA) and 15% trichloroacetic acid (TCA) in 0.25 N HCl was added. Thus, the reaction volume was 5 mL. Such prepared samples were subsequently incubated at 100°C for 20 min to optimize the conditions for the reaction of malondialdehyde (MDA) with TBA. The identification of TBARS in blood samples was achieved via the measurement of extinction at a wavelength of 532 nm versus the baseline sample after a preceding centrifugation (2000 ×g/15 min/4°C). Lipid peroxidation is expressed in the method by the level of TBA-MDA colored compounds, since MDA is the main component of TBARS that reacts with TBA. Other TBA-positive complexes would have to be present in the sample at extremely high concentrations to interfere significantly with the TBA-MDA compounds, as the wavelength of 532 nm is the maximum of absorption for TBA combined with MDA. Moreover, to exclude lipid peroxidation during the assay, an antioxidant, butylated hydroxytoluene (BHT), was added to the sample prior to TCA precipitation. The existence of EDTA in the sample from the Vacuette tube additionally protected against this phenomenon [21, 22]. The assay range was found to be 7 × 10^−2^–120 nmol/mL of plasma and 10–180 nmol MDA/g of hemoglobin (Hb). The concentrations of TBARS were expressed in 10^-2 ^nmol MDA/mL of plasma (TBARSpl) and nmol MDA/g of Hb (TBARSer). The CAT activity was estimated via the Beers and Sizer method [23]. The principle of the method is based on a decrease in the absorbance (*λ* = 240 nm) of a hydrogen peroxide (H_2_O_2_) solution. H_2_O_2_ is decomposed by the enzyme; thus, the decrease in absorbance is directly proportional to the CAT activity which was expressed in 10^4^ international units per g of hemoglobin (10^4^ IU/g Hb) [23]. The detection limits (DL) were between 15 × 10^4^ and 150 × 10^4^ IU/g Hb. The GPx activity was determined in accordance with the method described by Paglia and Valentine [24]. The method is based on the decomposition of H_2_O_2_ by GPx with the simultaneous oxidation of reduced glutathione. Oxidized glutathione is then reduced in a reaction catalyzed by glutathione reductase. A coenzyme in this reaction, reduced nicotinamide adenine dinucleotide phosphate (NADPH), is converted into an oxidized form (NADP^+^) and induces a change in the absorbance of light (*λ* = 340 nm) [24]. Since hydrogen peroxide is a catalase substrate as well, sodium azide (Na_3_N) was added to block this enzyme [24]. The GPx activity was expressed as U/g Hb and the sensitivity of the assay, expressed as DL, ranged from 2 to 36 U/g Hb. The SOD activity was measured using the Misra and Fridovich method [25]. The method is based on the inhibition of autoxidation of adrenaline to adrenochrome by the enzyme in alkaline environment, which induces a change in the extinction of the solution (*λ* = 480 nm) [25]. The SOD activity was expressed in U/g Hb and the DL were found to be 350–2050 U/g Hb.

### 2.4. Statistical Methods

The experimental data are shown as means ± standard deviations (SD) and were statistically analyzed using the analysis of variance (ANOVA) with post hoc analysis (Tukey's range test). Conformity to the normal distribution was determined on the basis of the Shapiro-Wilk test. The equality of variances was assessed using Levene's test. A *P* value of less than 0.05 was considered as a statistically significant difference.

## 3. Results

**Figure 1:**
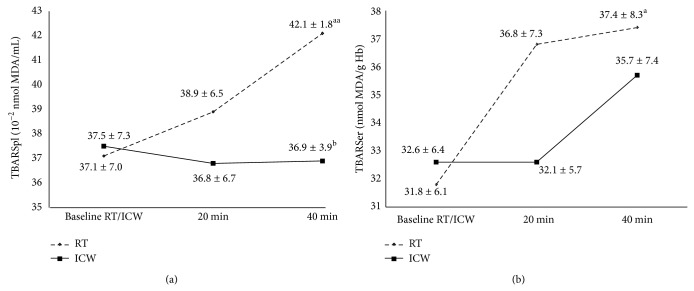
The TBARS concentration in blood plasma (TBARSpl) and erythrocytes (TBARSer) 20 and 40 min after recovery at room temperature (RT, 20°C) or in ice-cold water (ICW, 3°C) preceded by a 30 min aerobic exercise bout in healthy men. Statistically significant differences: versus baseline (^a^ *P* < 0.05, ^aa^ *P* < 0.01) and versus 40 min after the recovery at RT (^b^ *P* < 0.05). All data are shown as mean ± SD. TBARS, thiobarbituric acid reactive substances; MDA, malondialdehyde.

In the study, it was found that the TBARSpl concentration observed 40 min after the ICW recovery was lower by 14.1% than that observed when the subjects recovered at RT (*P* < 0.05; Figure 1(a)). However, the concentration of TBARSpl 20 min after the recovery in ICW versus 20 min after the recovery at RT did not differ in a statistically significant manner. Moreover, no statistically significant changes were found in the TBARSer concentration after the ICW recovery as compared with the RT recovery. No changes in either TBARSpl or TBARSer concentration were revealed after the ICW recovery versus their baseline levels (*P* > 0.05). On the other hand, after the exercise followed by the RT recovery, the levels of these lipid peroxidation products versus the baseline values increased in both blood plasma and erythrocytes (*P* < 0.01 and *P* < 0.05, resp.; Figure 1).

**Figure 2:**
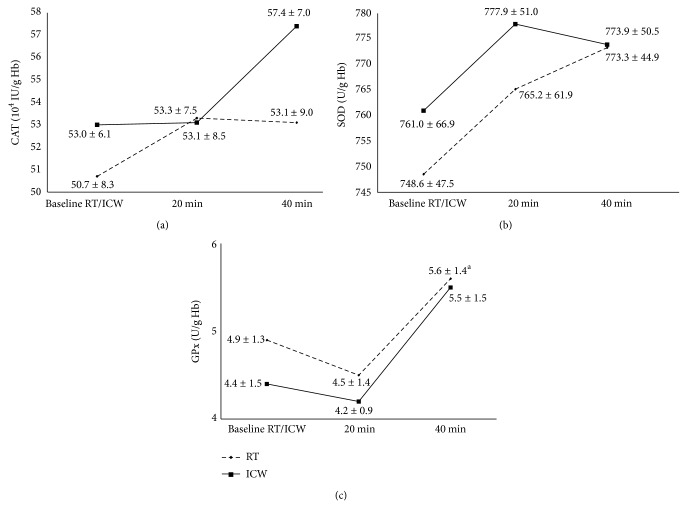
Erythrocytic antioxidant enzymes activities (CAT, SOD, and GPx) 20 and 40 min after recovery at room temperature (RT, 20°C) or in ice-cold water (ICW, 3°C) preceded by a 30 min aerobic exercise bout in healthy men. ^a^ *P* < 0.05, difference versus 20 min after the recovery at RT. No statistically significant differences were found in the CAT and SOD activities. All data are shown as mean ± SD. CAT, catalase; SOD, superoxide dismutase; GPx, glutathione peroxidase.

The only significant change in the antioxidant enzyme activities was a 24.4% increase in the GPx activity 40 min after the exercise/RT recovery as compared with the 20 min timepoint following this combination. Thus, the GPx activity after the exercise and RT recovery combination, as well as the exercise/ICW recovery combination, did not change compared to the baseline values (*P* < 0.05, Figure 2(c)). Moreover, the changes in the GPx activity in the entire experiment were similar between the RT and ICW recovery. Similar relationships of changes in the CAT activity between those two recovery types were also revealed. However, a tendency towards increase in the CAT activity 40 min after the exercise/ICW recovery as compared with the baseline was stronger than after the exercise/RT recovery (*P* > 0.05; Figure 2(a)). The SOD activity increased insignificantly 20 min after both recovery types compared to the baseline; however, after 40 min it decreased in the case of ICW recovery intervention and increased in the case of RT recovery intervention versus the 20 min timepoint (*P* > 0.05; Figure 2(b)).

The baseline values of oxidative stress parameters did not differ in a statistically significant manner between both study sessions/recovery types.

## 4. Discussion

The combination of the 30 min aerobic exercise and the ICW recovery may indicate that ice-cold water bath alleviates the lipid peroxidation process enhanced due to the exercise in healthy male subjects. The TBARSpl concentration 40 min after the exercise directly followed by the recovery in ICW was lower in comparison with the same timepoint in the exercise/RT recovery combination (*P* < 0.05). Moreover, there were no statistically significant changes in the TBARSpl and TBARSer concentrations after the exercise/ICW recovery compared to the baseline values, but the changes were observed after the exercise/RT recovery (*P* < 0.01 and *P* < 0.05, resp.; Figure 1). Probably, this could be the result of a decrease in the oxidative damage of lipids in blood plasma and the sarcolemma of skeletal muscles in the course of the exercise/ICW recovery intervention because, no doubt, ROS production has a strong positive correlation with intense exercise [13]. Ice-cold water bath, similarly to WBC, could also improve the mechanism of TBARS elimination: the catabolism of MDA (the major component of TBARS) in the liver [13]. However, Siems et al. revealed that even a single short-term immersion in ice-cold water (temperature between 1°C and 4°C) during winter swimming (air temperature between −1 and 5°C) is a source of ROS [6]. The authors reported that this ice-cold water exposure caused an increase in the oxidized glutathione (GSSG) concentration with a simultaneous decrease in the reduced glutathione (GSH) concentration in erythrocytes, as well as a significant decrease in the plasma uric acid concentration in winter swimmers versus healthy volunteers (control group) who had never performed winter swimming before that study [6]. It should also be mentioned that the baseline SOD and CAT activities were higher in the erythrocytes of the winter swimmers than in the volunteers from the control group [6]. The authors postulated that regularly repeated ice-cold water baths can help the human organism to adapt to oxidative stress; therefore, the baseline activities of the enzymes were higher in the winter swimmers [6]. They confirmed these findings also in their other study [26]. Thus, the increased activities of antioxidant enzymes prove increased ROS concentrations; however, this occurrence can also be profitable as it could constitute a stimulating change, not a damaging one [6, 26]. However, in the study, the decreased concentration of TBARSpl after exercise/ICW recovery versus exercise/RT recovery was not accompanied by a simultaneous increase in the antioxidant enzymes activities in such comparison (*P* > 0.05). Only a tendency for lower CAT activity 40 min after the exercise followed by the RT recovery than, at the same timepoint, after the exercise associated with ICW recovery was found (*P* > 0.05; Figure 2(a)). Other groups also described the adaption resulting from preexposure to lower doses of ROS. One of the groups revealed no changes in the activity of selected lysosomal enzymes in winter swimmers compared to non-winter swimmers after a single sauna bath [5], a source of ROS* per se* [27]. The authors explained the changes by the hardening of the organisms of winter swimmers, since the leakage of lysosomal enzymes may be induced by oxygen radical-mediated lipid peroxidation [5]. Therefore, the improved stability of lysosomal membranes probably resulted from an increase in the antioxidant capacity in winter swimmers [5]. The improvement of the performance of antioxidant defense in healthy people after long-term winter swimming was also found by Lubkowska et al. [15]. The authors demonstrated that a 5-month season of winter swimming improved antioxidant abilities in healthy men (*n* = 15) and, consequently, the subjects were more resistant to destabilizing effect of the WBC procedures [15]. However, in this study, ice-cold water bath was accompanied by aerobic exercise and the existence of exercise-induced oxidative stress was also revealed. It was found that, 40 min after the recovery at RT preceded by the 30 min aerobic exercise bout that is 70 min after the exercise bout, the TBARSpl concentration was significantly higher as compared with the baseline value (*P* < 0.01; Figure 1(a)). Similarly, TBARSer concentration changed in the subjects (*P* < 0.05). 40 min after the exercise/RT recovery the TBARSer concentration was higher by 17.6% in comparison with the baseline (*P* < 0.05; Figure 1(b)). An increase in the generation of ROS induced by physical exercise was also indicated by the increase in the GPx activity in the subjects' erythrocytes 40 min after the exercise/RT recovery as compared with the 20 min timepoint (*P* < 0.05; Figure 2(c)). However, the erythrocytic SOD and CAT activities did not change at all in response to the physical exercise, that is, both 20 and 40 min after the exercise bout followed by the RT recovery, as compared with the baseline (*P* > 0.05; Figures 2(a) and 2(b)). Moreover, the GPx activities after both the exercise/RT recovery and exercise/ICW recovery combinations did not change compared to the baseline values (*P* > 0.05). Therefore, the intensity of the exercise bout used in the study was probably not sufficient to cause strong oxidative stress. This confirms the subjects' assessment of the exercise bout as “moderate/somewhat hard” (Borg CR10; Table 1) [16]. The lack of statistically significant increases in the activities of these three enzymes 40 min after the exercise bout and recovery intervention, as compared to the baseline values, could also be caused by the fact that these enzymes are indicators of the first stage of ROS generation; however, the secondary indicators, TBARS, definitely increased [28]. Postexercise concentrations of ROS may be so high as to cause radical-mediated microinjuries of muscle fibers and connective tissues (articular cartilages, ligaments) which result in muscle pain, prolongation of recovery, and, consequently, a decrease in sports performance [29]. Thus, it seems reasonable to decrease the exercise-induced oxidative stress in order to improve performance, for example, via antioxidant supplementation [30]. However, there are also reports that indicate a completely opposite effect in the alleviation of the postexercise ROS levels [30]. It results from the fact that ROS are necessary for the proper muscle contraction during both rest and physical exercise [29]. Therefore, the literature recommends an adequate intake of antioxidants and balanced diet remains as the best approach to maintain the optimal antioxidant status in exercising individuals [30]. WBC is a procedure which balances the physiological effects of exercise and treats exercise-induced injuries [2]. The literature describes its effect as oxidant/antioxidant. It was found that a single session of WBC increased the antioxidant enzyme activities and lipid peroxidation in healthy untrained volunteers [12]. Another study in similar subjects showed that 10-day cryotherapy with one WBC session a day induced an increase in the total antioxidant status and the SOD activity [31]. Thus, WBC as an oxidant stimulates and, therefore, improves the antioxidant capacity in healthy untrained subjects. In turn, in elite athletes, WBC sessions used as pretraining stimulation during multiday training camps caused reverse effects: a decrease in both lipid peroxidation and the antioxidant enzyme activities [12–14]. Nevertheless, WBC is increasingly used in professional sports to facilitate the postinjury rehabilitation by reducing oxidative stress, inflammatory reactions, and pain in response to intense exercise [2, 12–14]. Thus, these inconsistent effects of WBC on the oxidant-antioxidant balance in humans probably help to maintain the most optimal ROS concentrations for human health and fitness, that is, during both rest and exercise. Similarly, the inconsistent results of the action of ice-cold water bath on the postexercise balance of the oxidation-reduction processes found in the study may suggest that bathing could also be successfully used for such purposes.

In conclusion, ice-cold water bath alleviated the level of lipid peroxidation after 30 min aerobic exercise on a cycloergometer in healthy men (*P* < 0.05). However, the rationale for the postexercise use of the 5 min head-out ice-cold water bath (3°C) for improving performance is still unknown, since, in this study, only a one-time procedure was used and the effect during long-term training bouts accompanied by ICW baths was not determined. Moreover, there were no changes in the antioxidant enzyme activities after the exercise bout accompanied by the recovery interventions compared to the baseline, as well as between the two types of postexercise recovery interventions. The temperature of ice-cold water might also be too low. Furthermore, in the literature, the long-term association of ICW baths with intense exercise is described inconsistently [9, 10]. Definitely, further studies are needed.

## 5. Conclusion

A 30 min aerobic exercise bout increases the generation of reactive oxygen species; therefore, it can disturb the oxidant-antioxidant balance in healthy men. However, a 5 min ice-cold water bath (3°C) decreases postexercise lipid peroxidation.

## Figures and Tables

**Table 1:** Characteristics of the study subjects. Values are given as mean ± SD.

Subjects' number	24
Age (years)	28.7 ± 7.3
BM (body mass, kg)	79.8 ± 10.2
BH (body height, cm)	181.9 ± 6.9
BF (body fat, %)	14.2 ± 4.6
TBW (total body water, %)	62.8 ± 3.3
MM (muscle mass, %)	44.9 ± 2.6
BMI (body mass index, kg/m)	24.1 ± 2.5
BS (body surface, m^2^)	2.0 ± 0.1
IPAQ (level of physical activity, MET·min/wk)	2574.3 ± 1000.6
VO_2max⁡_^1^ (maximum oxygen consumption, mL/kg/min)	42.1 ± 4.7
Borg CR10^1^ (rating of perceived exertion scale)	3.7 ± 1.1
VO_2max⁡_^2^ (maximum oxygen consumption, mL/kg/min)	44.3 ± 5.9
Borg CR10^2^ (rating of perceived exertion scale)^*^	3.5 ± 0.9

^1^Session 1 (no ice-cold water bath).

^2^Session 2 (ice-cold water bath).

^*^No statistically significant differences between VO_2max_ and Borg CR10 in both sessions.
